# X-ray cross-complementing family: the bridge linking DNA damage repair and cancer

**DOI:** 10.1186/s12967-023-04447-2

**Published:** 2023-09-07

**Authors:** Qiang Liu, Qiu Peng, Bin Zhang, Yueqiu Tan

**Affiliations:** 1 Hunan Cancer Hospital and the Affiliated Cancer Hospital of Xiangya School of Medicine, Central South University, 283 Tongzipo Road, Changsha, 410013 Hunan China; 2 NHC Key Laboratory of Human Stem Cell and Reproductive Engineering, School of Basic Medical Sciences, Institute of Reproductive and Stem Cell Engineering, Central South University, Changsha, 410078 Hunan China; 3 Clinical Research Center for Reproduction and Genetics in Hunan Province, Reproductive and Genetic Hospital of CITIC-Xiangya, Changsha, 410008 Hunan China; 4 Department of Histology and Embryology, Xiangya School of Medicine, Central South University, Changsha, 410013 Hunan China

**Keywords:** X-ray repair cross-complementary gene, XRCC, Cancer, DNA damage repair, Genomic instability

## Abstract

Genomic instability is a common hallmark of human tumours. As a carrier of genetic information, DNA is constantly threatened by various damaging factors that, if not repaired in time, can affect the transmission of genetic information and lead to cellular carcinogenesis. In response to these threats, cells have evolved a range of DNA damage response mechanisms, including DNA damage repair, to maintain genomic stability. The X-ray repair cross-complementary gene family (XRCC) comprises an important class of DNA damage repair genes that encode proteins that play important roles in DNA single-strand breakage and DNA base damage repair. The dysfunction of the XRCC gene family is associated with the development of various tumours. In the context of tumours, mutations in XRCC and its aberrant expression, result in abnormal DNA damage repair, thus contributing to the malignant progression of tumour cells. In this review, we summarise the significant roles played by XRCC in diverse tumour types. In addition, we discuss the correlation between the XRCC family members and tumour therapeutic sensitivity.

## Background

Genomic instability, a hallmark of cancer, ensues from a complex interplay involving DNA damage, tumour-specific flaws in DNA repair, and the inability to halt or impede the cell cycle prior to transmitting damaged DNA to daughter cells [1, 2]. Human DNA is exposed to tens of thousands of instances of damage each day, arising from both endogenous and exogenous factors, such as metabolites, ionising radiation (IR), ultraviolet (UV) light, and DNA damage resulting from replication errors [3–5]. Unrepaired DNA damage can significantly elevate the risk of various cancers, including breast, ovarian, prostate, and glioma, among others [6–9]. To maintain genome stability, cells adopt several measures to repair damaged DNA.

DNA damage repair (DDR) is one of the most critical biological responses in living organisms. The DNA repair pathway is usually a multi-step, nonlinear reaction involving a series of repair factors that work together in a time-series [10]. The DDR system contains five major repair pathways: base excision repair (BER), homologous recombination (HR), mismatch repair (MMR), nucleotide excision repair (NER), and non-homologous end-joining (NHEJ) [11]. Among all types of DNA damage, DNA double-strand breaks (DSB) are the most severe type of damage, and their efficient repair is essential for maintaining genome stability. There are two major DSB repair pathways in eukaryotes: HR and NHEJ [12, 13]. Mutations or aberrant expression of DDR-related genes result in compromised DNA damage repair functions, thereby reducing the capability of cells to repair damages caused by endogenous and exogenous stimuli. This fosters the accumulation of genetic alterations, ultimately leading to tumorigenesis [14]. DNA damage and abnormal DDR function not only contribute to tumorigenesis but also present opportunities and targets for tumour treatment. Many antitumour drugs operate in close association with the DNA damage and repair systems [15].

**Fig. 1:**
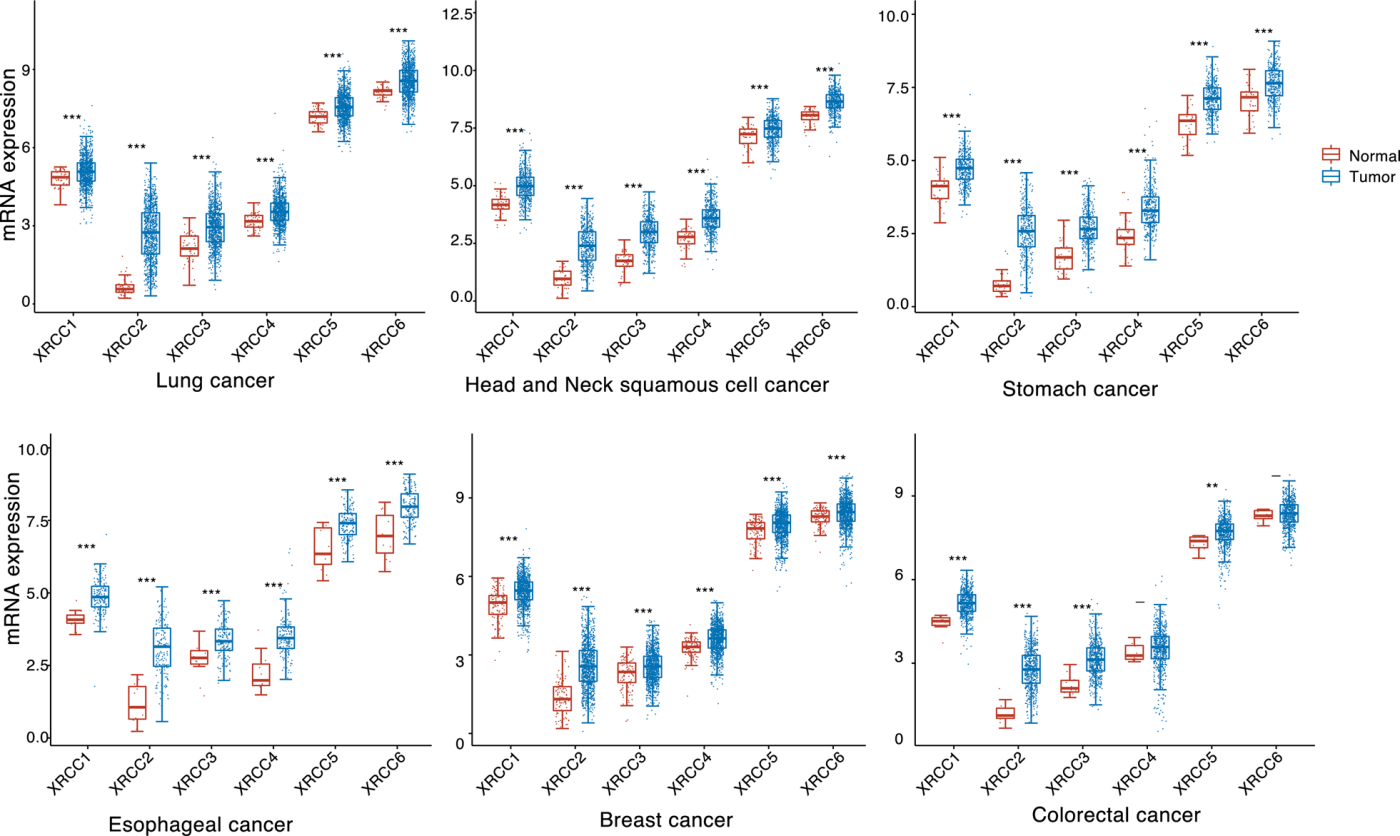
XRCC1-XRCC6 is abnormally expressed in a variety of tumours. The RNA-seq data of the tumours shown in the figure were obtained using The Cancer Genome Atlas (TCGA) database, and the expression levels of XRCC1–6 in tumour tissues and normal tissues were analysed, where the horizontal coordinates represent different genes and the vertical coordinates represent the gene expression distribution. Different colours represent different groups. *p < 0.05, **p < 0.01, ***p < 0.001

**Fig. 2:**
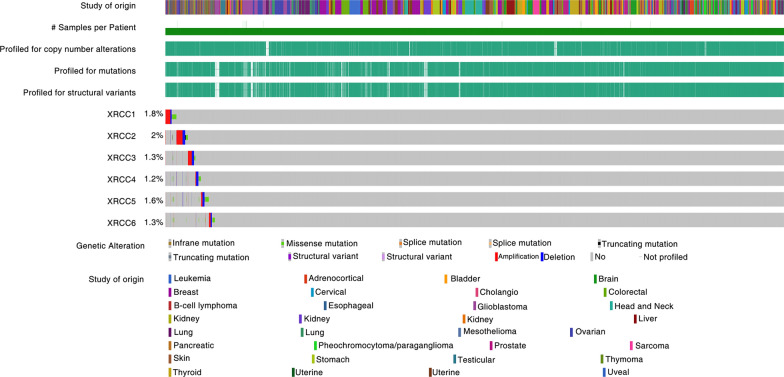
Genetic alterations of XRCC1–6 in pan-cancer. Analysis of XRCC1-XRCC6 mutations in pan-cancer using the cBioPortal database (https://www.cbioportal.org/)

The DNA repair system is a vast and intricate network closely intertwined with all aspects of life, yet it remains inadequately understood. To date, several repair-related genes have been identified; however, their specific functions are not well understood. Among these, X-ray cross-complementing (XRCC) genes are some of the most studied DNA repair genes, and their abnormal expression has been reported to be associated with the development of various malignancies [16–21]. The XRCC gene family comprises 11 main members (XRCC1–11), primarily responsible for maintaining chromosome stability by participating in DNA single-strand break repair [22, 23]. Among them, XRCC1–6 is a recognized member of the XRCC family, highly expressed in various tumour tissues and exhibiting multiple mutations in pan-cancer (Figs. 1 and 2). In addition, they play different biological functions in different cancer types (Table 1). In this review, we comprehensively elucidate the functions of the XRCC gene family in DNA damage repair, delving into their underlying mechanisms, and exploring their significant roles in tumour progression. In addition, we discuss the role of the XRCC gene family in the context of therapeutic sensitivities.

**Table 1:** The list of XRCCs and their biological functions in different cancer types

XRCCs	Cancer type	Biological function	Mechanism	References
XRCC1	Glioma	Proliferation, migration, invasion, and angiogenesis	Targeting MMP-2, cyclin D1, VEGF, and p16	[139]
XRCC1	Gastric cancer	Induction of cisplatin resistance	Targeting thioredoxin-like protein 1 (TXNL1)	[127]
XRCC1	Pancreatic cancer	Induction of apoptosis	Targeting base excision repair pathway	[140]
XRCC1	Clear cell renal cell carcinoma	Regulating tumour metastasis	Regulating the expression of MMP-2, MMP-9	[74]
XRCC1	Lung cancer	Tumour metastasis	Regulating the expressions of E-cadherin, N-cadherin, and vimentin	[113]
XRCC2	Hepatocellular carcinoma	Proliferation	Repairing mitochondrial DNA damage	[16]
XRCC2	Colorectal cancer	Cell growth, cell cycle progression, and apoptosis	Regulating bcl-2 expression	[141]
XRCC3	Glioma	Temozolomide resistance	Promoting DNA double-strand break repair	[18]
XRCC3	Esophageal squamous cell carcinoma	Improvement in radiotherapy effect	Promoting DNA damage repair and/or enhancing Telomere stability	[134]
XRCC3	Breast cancer	Induction of cisplatin resistance sensitization of chemotherapeutic	Stimulating Rad51-related recombinational repair	[132]
XRCC4	Retinoblastoma	Drugs development	Regulating DNA damage repair	[135]
XRCC4	Medulloblastomas	Tumour growth	Regulating Myc-family or Cyclin D2	[142]
XRCC5	Colorectal cancer	Cancer stemness and aggressiveness	Promoting cyclooxygenase-2 expression. In cooperation with p300	[143]
XRCC5	Colorectal cancer	Proliferation	Activating cyclooxygenase-2 expression and enhanced prostaglandin E2 production	[144]
XRCC6	Osteosarcoma	Proliferation and metastasis	Promoting β-catenin/Wnt signalling pathway	[145]
XRCC6	Hepatocellular carcinoma	Promotion of the transformation of precancerous hepatocytes and hepatocellular carcinoma development	Regulating the Wnt/β-catenin pathway	[146]
XRCC6	Hepatocellular carcinoma		Inducing an effective autophagic degradation	[17]

*MMP* matrix metalloproteinase, *VEGF* vascular endothelial growth factor, *XRCC* X-ray repair cross-complementing

## Structure and biological properties of the XRCC gene family

The XRCC family constitutes an essential group of DNA double-stranded break repair-related genes, responsible for encoding proteins involved in homologous recombination, which is indispensable for maintaining chromosomal stability and accomplishing DNA damage repair [24]. When DNA damage occurs, different XRCC genes participate in distinct DNA damage repair pathways. In the context of double-stranded DNA damage repair, XRCC2, 3, and 11 operate through the HR pathway, whereas XRCC4, 5, 6, and 7 operate through the NHEJ pathway [23]. Notably, among the 11 members of the XRCC family, the probability of XRCC7 (PRKDC), XRCC8, XRCC9 (FANCG), XRCC10, and XRCC11 (BRCA2) belonging to this family remains controversial [22].

**Fig. 3:**
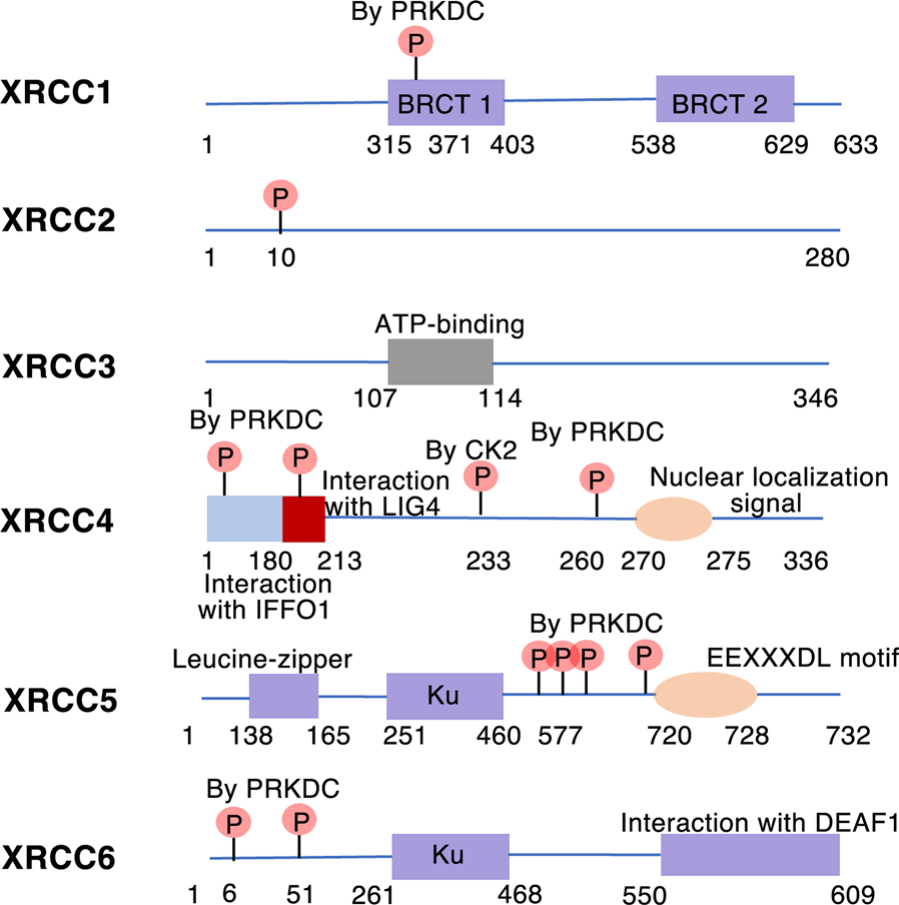
List and domain structures of XRCC1–6

*XRCC1* is located on chromosome 19q13.2–13.3, exhibits a total length of approximately 33 kb and contains 17 exons [25]. The *XRCC1* encodes a protein with three functional domains: the N-terminal domain, the BRCA1 carboxyl-terminal (BRCT) I domain, and the C-terminal BRCT II domain (Fig. 3), which interact with DNA polymerase beta, DNA ligase III, and poly(ADP-ribose) polymerase (PARP) to form a complex that acts as a “scaffolding protein” in the base excision repair process [23]. Human *XRCC1* was the first isolated mammalian repair gene reported to be associated with the repair of DNA damage caused by ionising radiation. In 1990, *XRCC1* was cloned by Thompson et al. from the gene library of EM9 cells [26]. In EM9 cells, the DNA ligase activity is reduced. Exposure to ionising radiation or ethyl methanesulfonate (EMS) led to impaired DNA strand breakage ligation and an elevated frequency of sister chromatid exchange (SCE). However, the introduction of XRCC1 rectified the deficiency in the DNA repair capacity of this particular cell line.

Human *XRCC2* is located at 7q36.1 and contains three exons [27]. XRCC2 is a newly discovered member of the RecA/Rad51 family of recombinant repair proteins. It is highly conserved in mammals and humans and encompasses the characteristic ATP-binding region typical of the Rad51 family [28]. The functions of XRCC2 include recruitment of the core protein Rad51 to the broken end of DNA, enhancement of Rad51 activity, maintenance of chromosome stability, and repair of DNA damage [29]. The loss of *XRCC2* expression can result in a defect in the core protein RAD51, leading to a significant reduction in the homologous recombination repair (HRR) function, particularly concerning DNA double-strand breaks. As a consequence, DNA damage cannot be effectively and timely repaired, giving rise to a considerably increased risk of chromosomal aberrations and abnormal chromosomal separation [30].

Human *XRCC3* is located on chromosome 14 q32.3, and the protein it encodes is involved in the recombination repair process of DNA double-strand breaks. The function of XRCC3 was first identified in irs1SF cells, a Chinese hamster ovary (CHO) cell line. Transfection of the cloned XRCC3 cDNA into irs1SF cells significantly improved chromosomal instability and reduced the sensitivity of irs1SF cells to various mutagens [31]. Liu et al. sequenced *XRCC3* and found homology with RAD51, a repair and recombination gene in eukaryotic cells; they further demonstrated the interaction between the two encoded proteins through a series of basic experiments. This indicates that the XRCC3 protein belongs to the RAD51-related protein family and plays a key role in the homologous recombination process, essential for preserving chromosome stability and repairing DNA damage [32].

Human *XRCC4* is located on chromosome 5q11.2–13.3 and encodes a 336 amino acid protein (Fig. 3). It exhibits a spherical N-terminal head structural domain comprising seven peptide chains folded into a flared β-barrel, which is further connected to a long helix tail. The process of polymerisation involves the association of the two head regions and the initial segments of their helix tails [33]. XRCC4 is an important NHEJ regulatory protein that directly interacts with Ku70/Ku80 in the repair pathway by preventing the degradation of free damaged DNA ends [34, 35]. XRCC4 can form a complex with DNA ligase IV and XLF, and then form an elastic link between Ku70/Ku80 and DNA ligase IV, guiding the damaged DNA ends to join each other, so that DNA can be repaired [36, 37].

Human *XRCC5* is located at 2q33–34 and encodes a 732-amino acid protein with a molecular mass of approximately 86 kDa [38] (Fig. 3). XRCC5, also known as Ku80, together with XRCC6 (Ku70) constitutes the XRCC5/XRCC6 heterodimer (Ku80/Ku70), which is a DNA-dependent protein kinase complex [37, 38]. The XRCC5/XRCC6 dimer binds to DNA double-stranded break ends and serves as an essential component of DNA nonhomologous end-joining repair [39].

## Single nucleotide polymorphisms (SNPs) in the XRCC family and tumour susceptibility

Single nucleotide polymorphisms (SNPs) are alterations in DNA sequence that are caused by variations in a single base at the genomic level. As the most common form of genetic variation, SNPs are commonly found in the human genome and constitute more than 90% of all variations in human genomic DNA, with an average of one genotypic polymorphic SNP per thousand bases [40, 41]. SNPs may be found in both the coding and non-coding sequences of genes. SNPs located in the coding regions of genes, specifically those genes encoding immune response factors, have the potential to impact differences in gene expression or alter the structure of proteins they encode [42, 43]. Numerous studies have highlighted the potential function of SNPs, such as their impact on gene or protein modifications, promoter activity, and the modification of transcription factor binding sites. Moreover, SNPs can also influence the subcellular localisation of RNA and/or proteins. In addition, SNPs are associated with certain human traits and can influence an individual’s susceptibility to specific diseases. Therefore, conducting an in-depth study of disease-associated SNPs and disease-susceptibility genes, along with analysing their functions, can significantly improve disease prevention strategies [44–47]. SNPs in the XRCC family of proteins play a significant role in causing individual variations in DNA damage repair ability, which in turn determines an individual’s susceptibility to tumours. Consequently, it is imperative to investigate genetic polymorphisms and tumour susceptibility and to explore specific molecular markers for the early diagnosis and treatment of tumours (Table 2).

**Table 2:** The relationship between SNPs of XRCC1–6 and tumour

Genes	Variants	Position	Cancer types/functions	References
XRCC1	G>C	c.1517	Increased risk of hepatocellular carcinoma development	[147]
	Arg399Gln	exon 10	Genetic biomarker of squamous cell carcinoma of the head and neck	[148]
	Arg399Gln	exon 10	Increased childhood risk of acute lymphoblastic leukemia	[149]
	G>A	c. 1196	Influence of colorectal cancer on the clinical outcomes of patients	[150]
XRCC2	R188H	rs3218536	Influence breast cancer risk and survival	[151]
	C>T	rs718282	Increased the cancer risk of endometrial cancer	[152]
XRCC3	Thr241Met	rs861539	Associated with the survival of glioblastoma multiforme patients	[153]
	A>G	rs1799796	Increased risk of prostate cancer	[154]
	A>G	rs1799794	Modulates the risk of head and neck cancer	[155]
XRCC4	G>T	c.1394	Associated with breast cancer development	[65]
	S110P	rs79561451	Influence the susceptibility of individuals to breast cancer	[156]
	A>G	rs1805377	Genetic markers of hepatocellular carcinoma	[157]
XRCC5	G>A	rs207906	Increased susceptibility to leukaemia	[158]
XRCC6	C>G	c.-1310	Associated with breast cancer risk and oestrogen exposure	[159]

*SNP* single nucleotide polymorphisms, *XRCC* X-ray repair cross-complementing

Extensive research on XRCC1 SNPs has unequivocally established their correlation with tumour risk, treatment response, and survival outcomes in diverse malignancies, including lung cancer and gastric cancer [48–51]. Several SNPs have been detected within the coding region of *XRCC1* that result in corresponding amino acid changes in the encoding protein. The C→T base transition in exon 6 of *XRCC1* results in the conversion of the amino acid encoded by codon 194 from Arg to Trp, leading to the formation of the XRCCl Arg194Trp gene polymorphism; the G→A base transition in exon 10 of *XRCC1* results in the conversion of the amino acid encoded by codon 399 from Arg to Gln, resulting in the formation of Arg399Gln gene polymorphism. Furthermore, the G→A base transition in exon 9 at position 27,466 results in the formation of Arg280His gene polymorphism [52]. *XRCC2* gene polymorphisms can potentially lead to alterations in the primary structure of XRCC2 or abnormal protein expression, resulting in impaired repair of DNA damage and increased susceptibility to cancer. Polymorphisms in *XRCC2* are associated with the development of various cancers, including lung, gastric, cervical, colon, breast, and others. Gok et al. reported that the Arg188His locus polymorphism of XRCC2 was significantly associated with the development of gastric cancer. Furthermore, Perez et al. demonstrated that the rs3218536 locus polymorphism of *XRCC2* was substantially associated with the risk of cervical cancer pathogenesis. In addition, Sirisena and Kluzniak reported that SNPs in *XRCC2* are associated with the risk of breast cancer pathogenesis [53–57]. *XRCC3* possesses multiple SNPs, and certain *XRCC3* SNPs have been inextricably linked to tumorigenesis, cancer progression, and susceptibility to treatment. These SNPs have the potential to serve as molecular indicators for predicting tumorigenesis and prognosis [58]. Several studies have demonstrated that the Thr241Met SNP of *XRCC3* is associated with susceptibility to various cancers, including lung, bladder, endometrial, and laryngeal cancers [59–63]. The SNP of *XRCC4* G1394T has been reported to be associated with colorectal carcinogenesis and susceptibility to lung and prostate cancer [64]. Furthermore, the c.1394G>T SNP in *XRCC4* is associated with the development of breast cancer in Filipinos [65]. This study suggests that SNPs of *XRCC5* are associated with the development and progression of various tumours. Liu et al. observed that rs828704, rs3770502, and rs9288516 SNPs in *XRCC5* are associated with an increased risk of glioma susceptibility [66]. Hayden et al. observed that individuals carrying the TT genotype exhibited a reduced risk of myeloma compared with those carrying the *XRCC5* rs2440 CC genotype [67]. The structure and function of *XRCC6* are regulated by multiple SNPs and are closely associated with the development and progression of several tumours. Numerous studies have reported that SNPs of *XRCC6* are associated with genetic susceptibility to various cancers, including head and neck, bladder, lung, kidney, prostate, oral, and gastric cancers [68–71]. In addition, *XRCC7* SNP at allele 3434Thr has been reported to be associated with the risk of thyroid cancer in Iranian patients [72].

## Role of XRCC in tumour metastasis

Metastasis refers to the process by which malignant tumour cells spread and establish secondary growths at distant sites from the primary tumour. The dissemination occurs through various means, including the lymphatic vessels, blood vessels, or body cavities from the primary site. Metastasis of malignant tumours is a major cause of death in cancer patients and a crucial factor affecting patient prognosis [73]. The XRCC family has been reported to regulate tumour metastasis by employing a variety of mechanisms. For instance, *XRCC1* is expressed at low levels in clear cell renal cell carcinoma (ccRCC) tissues in contrast to normal tissues. The ccRCC tissues with low *XRCC1* expression exhibit a positive correlation with lymph node metastasis and are associated with an unfavourable prognosis. Mechanistically, XRCC1 inhibits tumour cell invasion and metastasis by regulating the expression of tissue inhibitors of matrix metalloproteinase-2 (TIMP-2) and TIMP-1, leading to the suppression of the expression of metastasis-related markers matrix metalloproteinase-2 (MMP-2) and MMP-9 [74]. Additionally, the inhibition of *XRCC1* expression is associated with the progression of primary and metastatic melanoma [75]. The meta-analysis conducted by Bashir et al. revealed a significant downregulation of *XRCC2* in breast cancer tissues as opposed to non-cancerous healthy tissues. They also observed a significant correlation between *XRCC2* expression, lymph node status, and metastatic status in patients with breast cancer. These findings suggest that dysregulation of *XRCC2* in breast cancer could be utilized as a predictive indicator for lymph node metastasis and may serve as a therapeutic role in patients with breast cancer who are at risk of metastasis [76]. In colorectal cancer, the Thr241Met polymorphism of *XRCC3* is associated with time-to-metastasis and may potentially play a biological role in accelerating the metastatic process [77]. In breast cancer, scoring XRCC4 expression using immunohistochemistry has proven to be effective in predicting postoperative breast cancer metastasis. In addition, the combined diagnosis of XRCC4, PARP1, and excision repair cross-complementation group 1 (ERCC1) has demonstrated considerable predictive capability in assessing the risk of breast cancer metastasis [78]. *XRCC5*, a downstream gene of miRNA-188-5p, was reported to be upregulated in glioma samples. In contrast, miRNA-188-5p was down-regulated in these samples, and patients with glioma exhibiting low miRNA-188-5p expression levels showed higher rates of distant metastasis. In addition, it is observed that miRNA-188-5p regulates glioma cell metastasis by suppressing *XRCC5* expression [79]. In hepatocellular carcinoma, there is a positive correlation between *XRCC5* expression level and the migration and invasion abilities of hepatocellular carcinoma cells. Inhibition of *XRCC5* expression leads to a significant reduction in the migration and invasion abilities of hepatocellular carcinoma cells. Additionally, high *XRCC5* expression is associated with tumour size, microvascular invasion, and lower overall survival time in the clinical samples of patients with hepatocellular carcinoma. Mechanistically, XRCC5 regulates the expression of CTNNB1 (beta-catenin 1) and MMP9, which are key downstream target molecules of the Wnt/β-catenin signalling pathway. Through this regulatory function, XRCC5 promotes the progression of hepatocellular carcinoma [80]. Luo et al. reported that testicular expression 10 (TEX10) may potentially regulate cancer cell proliferation and metastatic processes through XRCC6, thereby controlling the Wnt/β-catenin signalling pathway and DNA repair channels [81]. These data suggest that the XRCC gene family plays an crucial role in tumour metastasis via multiple mechanisms.

## Role of XRCC in tumour immunity

At present, tumour immunotherapy is the most promising strategy for cancer treatment. It is used to treat tumours by harnessing the body’s immune system, enabling it to actively combat tumours, eradicate tumour cells, and establish sustained immune memory. Unlike targeted therapy, which focuses on specific targets, immunotherapy eliminates tumour cells by activating the body’s immune system and utilising immunoactive substances and immune cells produced by the body [82, 83]. Several immune checkpoints associated with tumour immunity have been identified, including cytotoxic T-lymphocyte antigen 4 (CTLA-4), programmed death 1 (PD-1), programmed death ligand 1 (PD-L1), T-cell immunoglobulin and mucin-domain containing protein-3 (TIM3), and lymphocyte activating 3 (LAG3), among others [84–87]. Damaged DNA repair and associated genomic instability not only elevate mutagenicity and oncogenicity but also augment the neoantigenic load on the surface of tumour cells, thereby increasing their immunogenicity [88, 89]. The XRCC family is closely associated with tumour immunity.

In colorectal cancer samples, mutations of *XRCC1* were significantly correlated with adenomas. Aberrant *XRCC1* expression and mutations contribute to adenoma carcinogenesis. Moreover, PD-1/PD-L1 expression and CD4+ intraepithelial lymphocytes (IELs) are associated with tumour progression in patients possessing the wild-type *XRCC1*, suggesting that *XRCC1* expression is correlated with patient survival, tumour-infiltrating lymphocytes, and immune marker expression [90]. Using bioinformatics analysis, Li et al. observed that in breast cancer XRCC2 and XRCC3 are associated with the infiltration of immune cells, such as B cells, CD4+ T cells, CD8/CD4+ T cells, neutrophils, and dendritic cells, as well as the prognosis of patients with breast cancer [91]. In head and neck, lung and cervical cancers, the methylation status of *XRCC3* is associated with the expression of immune checkpoint molecules and inflammatory markers [92]. Guo et al*.* reported that retinoic acid-inducible gene I (RIG-I) can potentially be recruited to double-strand breaks (DSB) and inhibit NHEJ. Mechanistically, RIG-I hinders the formation of the XRCC4/LIG4/XLF complex on DSB by interacting with XRCC4, thereby disrupting DNA repair and rendering cancer cells sensitive to radiation therapy. XRCC4 enhances RIG-I oligomerization and ubiquitination to promote RIG-I signalling, thereby inhibiting RNA viral replication in host cells, indicating the crucial role played by XRCC4 in the innate immune response [19]. The cGAS-STING pathway has emerged as a potential mechanism for the induction of inflammation-mediated tumorigenesis [93, 94]. Qi et al. reported that XRCC5 and XRCC6 are associated with the cGAS-STING pathway. Overexpression of *XRCC5* and *XRCC6* was significantly associated with the clinical stage and pathological grade of hepatocellular carcinoma. Moreover, they observed a significant correlation between the expression of *XRCC5* and *XRCC6* and the infiltration of B cells, CD4+ T cells, CD8+ T cells, macrophages, neutrophils, and dendritic cells in hepatocellular carcinoma [95]. In addition, the toll-like receptor 4 (TLR4)-mediated lack of immune activity inhibits the expression of *XRCC5* and *XRCC6* in response to damage by the carcinogen diethylnitrosamine (DEN). This effect leads to the impairment of DNA repair, facilitating the transformation of precancerous hepatocytes and the progression of HCC. In contrast, *XRCC6* expression prevents the development and progression of HCC by restoring the cellular senescence response and activating the immune network, thereby inducing efficient autophagic degradation, scavenging accumulated reactive oxygen species (ROS), reducing DNA damage, and attenuating proliferation [17, 96].

## Role of XRCC in tumour metabolism

The abnormal metabolism of tumour cells is an important feature of tumours. As normal cells gradually develop into tumour cells, they acquire several hallmark capabilities. Abnormal alterations in energy metabolism are one of the primary hallmarks of malignancy [97]. Tumour cells perform several biosynthetic processes and metabolic activities in a metabolic reprogramming manner, providing energy and multiple substrates to support their rapid proliferation and survival [98]. The activation of oncogenes or inactivation of tumour suppressors drives the metabolic reprogramming of cancer cells, and the XRCC family plays a critical role in the tumour metabolic process.

In a recent study, Anurag et al*.* observed that proteomic analysis of pretreatment patient biopsies uniquely revealed metabolic pathways associated with drug resistance, including oxidative phosphorylation, lipogenesis, and fatty acid metabolism. Interestingly, proteogenomic analysis of somatic copy number aberrations identified a resistance-associated deletion in 19q13.31–33, which corresponded with the location of *XRCC1* [99]. Aldehyde dehydrogenase 2 (*ALDH2*) is also involved in lipid metabolism. Chen et al. found that the interaction between the base excision repair proteins, XRCC1 and ALDH2, was indicative of overall survival in patients diagnosed with lung and liver cancer [100]. Folic acid metabolism is associated with the efficacy of platinum compounds [101, 102]. Folate metabolism involves DNA methylation mediated by the enzymes, tetrahydrofolate methylene reductase (MTHFR) and methionine synthase (MTR). Polymorphisms in *XRCC1* and folate metabolism genes can affect the prognosis of patients with non-small cell lung cancer [103]. In addition, polymorphisms in DNA repair genes (including *XRCC1*, *XRCC2*, and *XRCC3*) and steroid metabolism genes in patients undergoing prostate cancer radiotherapy are associated with clinically advanced toxicity [104].

## Role of XRCC in autophagy

Autophagy is a process by which self-damaged organelles and proteins are separated into autophagic vesicles and transported to lysosomes for catabolism [105]. Autophagy is closely associated with various diseases and plays a complex role in tumours. Particularly, autophagy plays an oncogenic role in early-stage tumours. Additionally, stressors such as nutritional deficiency, DNA damage, and cytotoxic effects can potentially induce cellular autophagy and promote malignant tumour progression in advanced-stage tumours or during antitumour therapy. Recent studies have shown that autophagy plays a dual regulatory role in promoting and inhibiting tumour cell growth; thus, targeting autophagy may significantly affect the efficacy of antitumour therapy [105].

Ma et al. conducted a comprehensive analysis including a cohort of 47 patients with advanced or metastatic oesophageal cancer who underwent next-generation sequencing (NGS) between May 2017 and February 2020. This study resulted in the identification of 227 mutated genes. Among them, *XRCC1* exhibited a substantial number of mutations and was associated with autophagy [106]. Demirbag-Sarikaya et al. observed that the autophagy-related molecule autophagy-related protein 5 (ATG5) interacts with both XRCC5 and XRCC6. This interaction is primarily mediated by XRCC6. They also found the interaction to be dynamic and enhanced under genotoxic stress. Moreover, they found that the interaction between ATG5 and XRCC6 is essential for DNA repair and effective recovery from genotoxic stress. These results demonstrate a novel, direct, dynamic, and functional interaction between ATG5 and XRCC6, which are proteins that play critical roles in DNA repair under genotoxic stress conditions [107]. In addition, Wang et al. showed that the restoration of immunity supporting hepatocyte senescence and autophagy through XRCC6 repair of DNA damage reverses the progression of TLR4-deficient deteriorating hepatocellular carcinoma [17, 96].

## The influence of non-coding RNAs on XRCC

Non-coding RNA (ncRNA) is an emerging biomarker that exhibits correlations with tumorigenesis and possesses oncogenic or tumour-suppressing properties. It can be detected in serum, plasma, and other biological fluids, making it a promising therapeutic and prognostic target for tumours, due to its non-invasive nature traumatic, high sensitivity, and specificity [108–110]. The ncRNAs, including long ncRNAs (lncRNAs), microRNAs (miRNAs), and circular RNAs (circRNAs), are extensively involved in tumour pathogenesis. The ncRNAs play a pivotal role in the biological processes of tumours by regulating cell growth and survival, EMT and metastasis, maintenance of tumour stem cells, metabolism, autophagy, chemoresistance, and angiogenesis [111, 112]. Several studies have reported that ncRNAs modulate tumour progression by regulating *XRCC*. In lung cancer, the circular RNA FLNA acts as a sponge for miR-486-3p and promotes tumour cell proliferation, migration, and invasion by regulating *XRCC1* expression [113]. Li et al. observed that miR-3940-5p enhances homologous recombination repair after DSB by down-regulating *XRCC2* expression [114]. In oesophageal cancer, microRNA-127-3p enhances the chemosensitivity of phenanthroline-dione derivatives by targeting XRCC3 [115]. In glioma cells, the long non-coding RNA SBF2-AS1 acts as a ceRNA for miR-151a-3p, which in turn regulates the expression of *XRCC4*, thereby enhancing DSB repair [116]. Furthermore, in hepatocellular carcinoma cells, lncRNA NIHCOLE promotes the ligation efficiency of DSB by regulating XRCC4 [117]. CircXRCC5 acts as a sponge for miR-490-3p and regulates the expression of the downstream target gene, XRCC5, thereby activating CLC3 transcription and promoting glioma progression [118]. In breast cancer, miR-623 inhibits cell proliferation, migration and invasion by targeting XRCC5 by downregulating cell cycle protein-dependent kinases and inhibiting phosphatidylinositol-3 kinase (PI3K)/Akt and Wnt/β-Catenin pathways [119]. The correlation between microRNA-379-5p and premature ovarian insufficiency has been reported to be mediated by PARP1 and XRCC6 [120].

## Role of XRCC in tumour therapeutic sensitivity

Platinum-based combination chemotherapy represents the first-line standard of care for numerous types of tumours. The primary mechanism of action of platinum-based drugs is the formation of platinum–DNA adducts binding to guanine, adenine, and cytosine on DNA. This process leads to the creation of inter-strand or intra-strand DNA cross-links, ultimately causing DNA damage and cell death [121]. Differences in DNA repair ability directly lead to inter-individual differences in the sensitivity of tumour cells to DNA-related cytotoxic drugs [122]. Therefore, the relationship between DNA repair genes and tumour susceptibility to platinum-based chemotherapy may be crucial for guiding individualised clinical treatments. Similarly, the biological mechanism of killing tumour cells by radiation therapy is primarily based on direct genomic damage caused by radiation, resulting in the loss of the proliferative ability of tumour cells. Therefore, the clinical effect of radiation therapy depends on the responsiveness of tumour cells to radiation damage and their ability to repair the damage. However, tumour cells are highly capable of damage repair and can selectively recognise damage and initiate repair pathways, leading to tumour cell tolerance to radiation therapy and other antitumour drugs. Studies have demonstrated that DNA damage repair mechanisms protect tumour cells from radiation therapy-induced cell death, indicating that repair pathway proteins may play a potential role in enhancing tumour cell radiosensitivity. Exploring new approaches to more effectively inhibit repair proteins is crucial for enhancing tumour radiosensitivity [123].

DNA repair ability is associated with the Gln399Arg polymorphism in *XRCC1*. Patients with non-small cell lung cancer polymorphism may potentially be resistant to platinum [50, 124]. In a study involving 195 patients with epithelial ovarian cancer, it was observed that 45% of patients with XRCC1-positive tumours were resistant to platinum drugs. In contrast, only 17% of patients with XRCC1-negative tumours were resistant to platinum drugs. These findings suggest that XRCC1 has clinical significance as a predictor of resistance to platinum therapy in patients with ovarian cancer [125]. Xu et al. reported that the methylation level of H3K4 is significantly reduced in drug-resistant cells. JIB-04, a chemical inhibitor of H3K4 demethylase, restores the methylation of H3K4, blocks the co-localisation of XRCC1 and phosphorylation of H2AX (γH2AX), and ultimately improves drug sensitivity. They also found that the expression level of KDM5B was significantly elevated in drug-resistant cells. Knockdown of KDM5B elevates the methylation level of H3K4, which hinders the localisation of XRCC1 at the DNA damage site, resulting in heightened sensitivity [126]. Furthermore, in the context of gastric cancer, it has been reported that XRCC1 expression was significantly elevated in cisplatin-resistant cells, and it independently promoted cisplatin resistance. Irinotecan, another chemotherapeutic agent that induces DNA damage, was used to treat patients with advanced gastric cancer who experienced progression on cisplatin therapy. Notably, irinotecan effectively inhibited XRCC1 expression, resulting in increased sensitivity of resistant cells to cisplatin [127].

*XRCC2* is indispensable for DNA repair following radiation damage. Radiation induces an abnormal increase in the expression level of *XRCC2* in lung cancer cells, which causes them to resist the damaging effects of radiation on tumour cell DNA. This results in the development of tumour resistance to radiotherapy [128, 129]. In glioblastoma, inhibition of *XRCC2* expression increases the radiosensitivity of tumour cells to radiation [130]. X-ray irradiation induces *XRCC2* expression in colorectal cancer cells and exhibits a dose- and time-dependent relationship between *XRCC2* expression and radiation exposure. Downregulation of *XRCC2* expression inhibits the proliferation of colorectal cancer cells and increases their sensitivity to radiation. In addition, gene silencing of *XRCC2* induces a decrease in the repair of radiation-induced cell damage, resulting in cellular arrest in the G2/M phase and increased apoptosis [131]. Expression abnormalities in *XRCC3* are associated with tumour resistance to DNA damage-inducing antitumour agents. XRCC3 induces cisplatin resistance in tumour cells by activating Rad51-related recombination repair and S-phase monitor activation and by reducing apoptosis [132, 133]. XRCC3 has been reported to protect glioma cells from temozolomide (TMZ)-induced cell death and cell cycle inhibition. In addition, *XRCC3* knockdown significantly reduces DSB repair after TMZ treatment, leading to increased drug sensitivity. This study confirms the importance of homologous recombination in conferring resistance to the methylating drug TMZ of glioma cells [18]. High XRCC3 expression is positively associated with resistance to radiotherapy in oesophagal squamous cell carcinoma (ESCC) and is an independent predictor of short disease-specific survival in patients with ESCC. Knockdown of *XRCC3* in ESCC cells significantly improved the efficacy of radiotherapy in both in vitro and in vivo analyses. XRCC3 overexpression significantly enhanced the resistance of ESCC cells to radiotherapy. Furthermore, the radiation resistance of XRCC3 was mainly dependent on enhanced homologous recombination, telomere stabilisation, and ESCC cell death reduction mediated by radiation-induced apoptosis and mitotic mutations [134]. Overexpression of ubiquitin-like with PHD and RING finger domains 1 (UHRF1) increases *XRCC4* expression Conversely, the downregulation of XRCC4 renders retinoblastoma cells sensitive to etoposide treatment, indicating that XRCC4 is a key mediator of drug sensitivity following UHRF1 consumption in retinoblastoma cells. Moreover, in retinoblastoma cells depleted of UHRF1, it was observed that the chromatin association of DNA ligase IV in response to acute DNA damage was significantly reduced. Functional complementation of XRCC4 in cells depleted of UHRF1 weakens drug sensitivity, indicating that the downregulation of XRCC4 in UHRF1-depleted cells impairs DNA repair, leading to a significant induction of apoptosis during treatment with genotoxic drugs [135]. Hori et al. investigated the relationship between NHEJ-related protein expression and the outcome of radiotherapy in oesophageal cancer. They employed immunohistochemical analysis of NHEJ-related proteins, including XRCC4, which holds promise as a potential predictive marker for assessing tumour radiosensitivity [136]. *XRCC5* knockdown significantly enhanced the sensitivity of glioma cells to TMZ, whereas XRCC5 overexpression led to TMZ resistance in cancer cells. Both in vitro and in vivo experiments have shown that TMZ treatment induces XRCC5 expression in TMZ-resistant cells [137]. Chen et al. reported that the quercetin-targeted radiation-induced ARv7-mediated circNHS/miR-512-5p/XRCC5 signalling pathway increases radiosensitivity in prostate cancer [138].

## Conclusions

Tumour development is the result of a complex interplay of various factors, and DNA damage is a significant contributor to this process. The XRCC gene family is a crucial group of genes involved in DNA damage repair responsible for maintaining the stability of genetic material and cellular function through their role in repairing DNA double-strand breaks and cross-link damage. Additionally, these genes play a significant role in ensuring the proper segregation of chromosomes during cell division. The XRCC family constitutes a group of susceptibility genes, and their polymorphisms are prevalent in the general population, exerting a substantial effect on tumorigenesis. An in-depth investigation of the correlation between XRCC gene polymorphisms and tumour development can help explore the interactions between related genes, as well as the interactions between genes and the environment. These investigations will substantially help in effectively formulating tumour prevention and treatment strategies, protecting the susceptible population to a larger extent, effectively reducing the incidence of tumours, and improving the cure rate of tumours. Although significant progress has been made, inconsistencies persist in the findings of several studies. Therefore, it is essential to increase the sample size and conduct a comprehensive population cohort study employing multivariate analysis of crucial prognostic factors, such as gender, age, smoking status, histopathological types, clinical stages, and treatment strategies. This approach enables the investigation of the correlation between gene polymorphisms and prognosis, as well as the interplay between genetic polymorphisms and environmental factors.

The mechanism of resistance to tumour radiotherapy and chemotherapy has been a popular research topic in the field of oncology. DNA oxygenation and alkylation damage caused by numerous DNA-damaging anticancer drugs can be repaired via the XRCC gene family-mediated pathways. Research on the XRCC gene family and chemotherapeutic drug sensitivity is of particular interest. Inhibition of the XRCC gene family expression can sensitise various anticancer drugs, suggesting the XRCC gene family has the potential to influence the efficacy of tumor therapy by affecting chemotherapy sensitisation. However, the functions of these genes are not fully understood, and their relationship with anticancer drug sensitisation requires further exploration.

## Data Availability

Not applicable.

## Abbreviations

XRCC: X-ray repair cross-complementary
IR: Ionizing radiation
UV: Ultraviolet
DDR: DNA damage repair
BER: Base excision repair
HR: Homologous recombination
MMR: Mismatch repair
NER: Nucleotide excision repair
NHEJ: Non-homologous end joining
DSB: DNA double-strand breaks
EMS: Ethyl methanesulfonate
SCE: Sister chromatid exchange
PARP: Poly(ADP-ribose) polymerase
SNP: Single nucleotide polymorphisms
ccRCC: Clear cell renal cell carcinoma
CTLA-4: Cytotoxic T-lymphocyte antigen 4
PD-1: Programmed death 1
PD-L1: Programmed death ligand 1
IELs: Intraepithelial lymphocytes
TLR4: Toll-like receptor 4
DEN: Diethylnitrosamine
ROS: Reactive oxygen species
ALDH2: Aldehyde dehydrogenase 2
MTR: Methionine synthase
NGS: Next-generation sequencing
ESCC: Esophageal squamous cell carcinoma
TMZ: Temozolomide
